# Spatial characteristics and determinants of traditional village distribution in Guizhou Province

**DOI:** 10.1371/journal.pone.0324275

**Published:** 2025-05-30

**Authors:** Qiuju Mao, Liang Xie, Lai Fan

**Affiliations:** 1 School of Culture and Arts Media, Guizhou University of Commerce, Guiyang, China; 2 College of Design and Engineering, National University of Singapore, Singapore, Singapore; 3 School of Architecture and Urban Planning, Beijing University of Civil Engineering and Architecture, Beijing, China; Central South University School of Architecture and Art, CHINA

## Abstract

This study employs a range of analytical techniques, including the geographical detector, kernel density estimation, imbalance index, geographical concentration index, and nearest neighbor index, all integrated with ArcGIS 10.8, to examine and illustrate the spatial distribution of 757 traditional villages across Guizhou, revealing an aggregated spatial distribution pattern of traditional villages, i.e., “one highly concentrated area and two secondary density clusters.” This pattern is influenced by both natural and socio-cultural factors, with socio-cultural elements such as road network density, GDP, and ethnic minority populations playing a more significant role than natural environmental factors. The results of geodetector analysis indicate that the interaction between these factors generally shows a nonlinear enhancement effect. Based on these findings, this study proposes four main strategies to preserve and enhance traditional villages: (1) establishing regional identities that reflect local ethnic characteristics; (2) improving village infrastructure to enhance accessibility; (3) implementing targeted protection and utilization strategies based on local conditions; and (4) industrial linkage, combining protection and development.

## 1. Introduction

Traditional villages are defined as communities that originated early, have maintained stable locations, and have persisted to the present day, continuing to serve their populations while embodying rich economic, historical, cultural, artistic, and social values [1]. The onslaught of rapid urbanization has led to several adverse phenomena, including disorganized human settlement construction, the decline of community culture, village hollowing and marginalization, and natural surrounding destruction [2]. Many traditional villages now face abandonment and disappearance threats. In December 2012, the Ministry of Housing and Urban-Rural Development of China initiated the formalized protection of these cultural assets by publishing the first list of Traditional Chinese Villages. To date, various national departments have released six batches of lists, encompassing a total of 8,155 villages [3]. Concurrently, they have issued and implemented relevant policy documents focused on maintaining these villages’ residential settings, cultural legacy, historical elements, and geographical configuration, while also enhancing their social functions and contemporary values. Particularly since 2017, as the Government advocates for “comprehensively advancing the rejuvenation of rural areas,” safeguarding and enhancing traditional villages has been recognized as an effective strategy for supporting rural economic development and enhancing ecological livability. Guizhou Province holds the second-highest count of traditional villages in the country, with 757 of them grouped into six batches [4]. Not only are these villages numerous and widely distributed, but they are also endowed with diverse resources spanning various ethnic cultures and types. Based on this, Guizhou Province also issued the “Regulations on the Protection and Development of Traditional Villages in Guizhou Province” at the end of 2024, clarifying the measures for the protection and development of traditional villages. Guizhou Province is a uniquely poverty-stricken area in western China. In 2024, its per capita GDP ranked the fourth lowest in the country, and 14 counties were listed as deeply impoverished counties. For example, the poverty incidence rate in Wangmo County is as high as 21.5%, and the poverty incidence rate in Rongjiang County is as high as 25.54%, etc. Moreover, these poverty-stricken areas are precisely the regions where ethnic minorities are relatively concentrated [5]. Therefore, analyzing the formation patterns, factors, and spatial features of traditional villages in Guizhou holds significant practical importance. This analysis not only aids in maintaining and conveying traditional villages’ cultural, artistic, and historical values but also provides a foundation for relevant policy formulation and development planning. Such efforts strive to simultaneously safeguard traditional villages and revitalize rural areas.

Research on rural settlements has a long and rich history, with its origins traceable to the 1841 work by German geographer Kohl, titled “The Relationship between Human Traffic, Habitation, and Terrain.” This seminal publication systematically analyzed the interplay between settlement distribution and terrain features [6]. In the same year, Bunce initiated studies into the formation of rural settlements, positing that environmental and economic factors play significant roles in their development [7]. Roberts further explored the relationships between rural settlement distribution, landform, and transportation accessibility [8], while Crouch delved into rural village culture, underscoring the pivotal role of cultural connotations on village development [9]. A multitude of foreign scholars have lately adopted interdisciplinary approaches to examine the spatial layout of rural settlements, focusing on various aspects such as the living and productive activities of villagers [10], conservation of cultural heritage [11], village architecture [12], cultural landscapes [13], sociodemographic characteristics of villagers [14], the effects of population transfer and mobility on rural settlements [15], and the effect exerted by administrative divisions on rural settlements’ spatial distribution [16]. This broad and diverse body of research has significantly expanded the scope and depth of understanding in the field, providing valuable perspectives for traditional villages’ preservation and growth. Though studies on rural settlements in China began later than in many Western contexts, the urgency and significance of conserving traditional villages have propelled a growing body of scholars to engage in this area. Recent studies have focused on traditional villages’ layout and form [17,18], the identification and impact of characteristics in traditional villages [19,20], and preservation and enhancement strategy formulation [21]. Additional research has examined the human settlement environment within these villages [22] and tourism development models tailored to their unique contexts [23]. Methodologically, there has been a notable shift from primarily descriptive qualitative analyses toward more quantitative approaches, both in China and internationally. This includes the application of methods like buffer zone analysis [24], nearest neighbor distance measurements [25], kernel density analysis [26], and the use of geographical detectors [27].

Although studies on Chinese traditional villages have been ongoing for some time and have achieved notable results, they have predominantly concentrated on the more developed provinces, such as those along the eastern coastal regions, Yunnan, and Sichuan [28]. In the case of Guizhou Province, studies on the spatial morphology, factors affecting spatial formation, and traditional villages’ preservation and enhancement are relatively scarce. Most existing research has focused on heritage-related themes, including the cultural landscape [29], village aesthetics [30], and tourism development of specific villages [31], often employing a limited array of quantitative methods. Geographically, the emphasis has largely been on the southeastern part of Guizhou, with a significant concentration on Dong and Miao villages [32,33], resulting in a geographical imbalance in research coverage. Moreover, following the 2022 disclosure of the sixth collection of traditional villages, the scope of existing studies has predominantly encompassed the initial five batches, thus not fully addressing all currently recognized traditional villages. This study seeks to address and expand the scope.

The objective of the study is to:

(1) Analyze the present state of traditional villages’ preservation and enhancement in Guizhou, focusing on their spatial distribution characteristics and conducting comprehensive research on their preservation and enhancement.
(2) Identify determinants of traditional village distribution in Guizhou Province, evaluate their positive and negative impacts as well as the impact degree, and assess the intensity of their interactions.
(3) Explore the process that traditional villages’ distribution pattern is formed in Guizhou Province.
(4) Propose specific conservation and enhancement plans for traditional villages in Guizhou Province in light of these influencing factors and the historical causes underlying their formation.

## 2. Materials and methods

### 2.1 Study area

**Fig 1:**
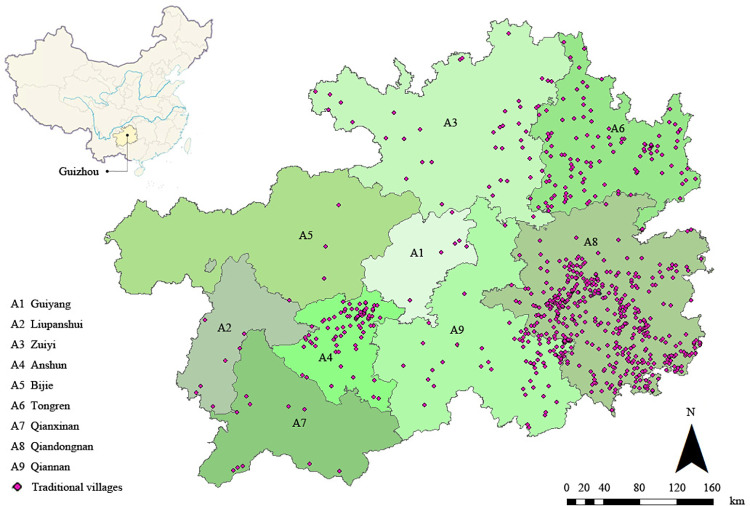
Distribution map of traditional villages in Guizhou. Note: The base map came from Natural Earth (http://www.naturalearthdata.com/).

Guizhou Province is positioned in the southwest region of China, with an area of about 176,200 square kilometers. The administrative structure of the province includes 88 county jurisdictions, three autonomous regions, and six cities at the prefecture level, which encompass 16 districts, 63 counties, and nine county-level cities. The landforms of the entire province can be summarized into three basic types: plateau mountains, hills, and basins. Among them, 92.5% of the area is composed of mountains and hills, making it the only province in the country without plains. In the northern part of the territory, there is the Dalou Mountain, and the Miaoling Mountain runs across the central and southern parts. In the northeastern border, there is the Wuling Mountain, which meanders from Hunan into Guizhou. In the western part, the Wumeng Mountain towers, and in Zhushi Township, Hezhang County, which belongs to this mountain range, the Jiucaiping has an altitude of 2900.6 meters, being the highest point in Guizhou [34]. The topography generally slopes steeply, rising in the west and descending towards the east. Elevations range from over 2,900 meters above sea level to below 300 meters, and the average altitude is approximately 1,100 meters. The region is endowed with abundant water resources, with the Wujiang River, Nanpan River, Beipan River, Qingshui River, Chishui River, among others, being the principal waterways [35]. The climate is subtropical and humid, characterized by distinct seasons, mild winters, and cool summers. The annual average temperature ranges between 14 and 16 degrees Celsius, and annual precipitation varies from 800 to 1,800 millimeters [36]. Moreover, Guizhou Province boasts a rich history and diverse traditional cultural characteristics, historically situated at the heart of the Miaojiang Corridor and typically categorized as a “raw Miao” area during the period of territorial reassignment and native governance. This region is inhabited by numerous ethnic minorities, preserving a vast number of distinctive traditional villages. To this day, Guizhou is home to 50 ethnic groups, 49 of which are minorities [37]. As of July 2024, the province has 757 traditional villages listed on the National Register of Traditional Chinese Villages, ranking it second in China for the traditional village count. These traditional villages of ethnic minorities, primarily established during the Ming and Qing dynasties, have developed their present layouts through repeated migrations, divisions, and reorganizations [38] (Fig 1).

### 2.2 Data sources

By leveraging the Gaode Map API interface, we employed Python web scraping techniques to acquire latitude and longitude data for 757 traditional villages across six batches in Guizhou Province. These data were subsequently imported into ArcGIS software for visualization, leading to the generation of a map depicting the traditional village distribution. Through spatial joining, traditional villages’ point features were linked with the administrative divisions of Guizhou Province, enabling the extraction of information on the number of traditional villages within each prefecture-level city. This information served as the basis for spatial analysis. The Geospatial Data Cloud provided additional data, including administrative divisions, elevation maps, municipal water systems, and transportation networks within Guizhou. The China Meteorological Administration’s website provided climate-related data, such as temperature and precipitation. Economic and demographic data for each administrative region originated from the government portal for Guizhou, the “Guizhou Statistical Yearbook (2023),” and the “Seventh National Population Census Bulletin of China.”

### 2.3 Research methods

In this study, an array of visualization and quantitative methodologies is employed to investigate traditional villages’ distribution characteristics in Guizhou, and to deeply explore the determinants of traditional villages. According to the existing list of traditional villages in Guizhou Province, the nearest neighbor index in ArcGIS 10.8 software was used for discrimination. The imbalance index was utilized to assess the equilibrium degree in the region’s traditional village distribution. The geographical concentration index was used for predicting traditional villages’ aggregation level at the municipal level. The results of these three models are mutually corroborative. Furthermore, the kernel density estimation method was used for data visualization, permitting us to intuitively analyze the distribution density and spatial variability with regard to traditional villages. Moreover, the factors influencing kernel density are quantified, and an index system of influencing factors is constructed. Subsequently, the Geodetector is employed. When an independent variable considerably impacts a particular dependent variable, independent and dependent variables display similar spatial distribution characteristics [39]. With the Geodetector, the influence degree of the above-mentioned data on traditional villages is detected. Subsequently, both single-factor and interaction-factor detection methods are employed to determine whether an interaction exists between two factors, as well as to assess the strength and direction of such an interaction, thereby examining the influencing factors of traditional villages’ spatial distribution characteristics (Table 1).

**Table 1:** Research methods.

Research Methods	Calculation Formula	Calculation Formula
Nearest Neighbor Index Method	$\begin{array}{c} {\bar{r}}_{E}=\frac{1}{2\sqrt{m/A}}=\frac{1}{2\sqrt{D}} \end{array}$	D denotes the density value of traditional villages’ spatial distribution, A the total land area of Guizhou, m the count of point elements of Guizhou’s traditional villages, $r_{E}$ and $\begin{array}{c} {\bar{r}}_{1} \end{array}$ the theoretical and actual nearest neighbor distance, respectively. R = 1, $\begin{array}{c} {\bar{r}}_{1} \end{array}$ = $r_{E}$, indicates a random distribution, R > 1, $\begin{array}{c} {\bar{r}}_{1} \end{array}$ > $r_{E}$,t, a uniform distribution, R < 1, $\begin{array}{c} {\bar{r}}_{1} \end{array}$ < $r_{E}$, an aggregated distribution.
	$\begin{array}{c} R=\frac{{\bar{r}}_{1}}{r_{E}}=2\sqrt{r_{1}D} \end{array}$	
Geographic Concentration Index	$G=\sqrt{\sum_{i=1}^{n}{\left(\frac{X_{i}}{T}\right)}^{2}}\times 100$	G represents the geographic concentration index value of traditional villages in Guizhou, with $X_{i}$ denoting the traditional village count in the i-th city among the nine cities in Guizhou; T the complete traditional village count; and n Guizhou’s city count. G value varies from 0 and 100. Higher G values signify a denser distribution, whereas a lower G suggests a more dispersed distribution.
Imbalance Index	$\begin{array}{c} S=\frac{\sum {\mathrm{\backslash nolimits}}_{i=1}^{n}Y_{i}-50\left(n+1\right)}{100n-50\left(n+1\right)} \end{array}$	n indicates the traditional village count, $Y_{i}$ the aggregate proportion of traditional villages by region, sorted in descending order. S ranges from 0 to 1, where S = 0 signifies an even distribution, and S = 1 a concentrated, clustered distribution.
Kernel Density	$\begin{array}{c} f_{n}\left(X\right)=\frac{1}{nh}\sum_{i=1}^{n}k\left(\frac{x-x_{i}}{h}\right) \end{array}$	$k\left(\frac{x-x_{i}}{h}\right)$ indicates the kernel function, $f_{n}\left(X\right)$ the kernel density function, $x\;and\;x_{i}$ the geographic coordinates, and h the bandwidth. A higher value suggests a denser distribution of traditional villages across Guizhou Province.
Geodetector	$\begin{array}{c} q=1-\frac{1}{{N_{\sigma }}^{2}}\times \sum_{h=1}^{l}N_{h}{\sigma_{h}}^{2} \end{array}$	q varies in the range of 0–1, with higher q values signifying stronger spatial variability of Y. h = 1,…, L represents the stratification of variable Y or factor X, N and $N_{h}$ the total region and the counts of units in stratum h respectively; ${\sigma_{h}}^{2}$ and $\sigma^{2}$ the variances within stratum h and the total region separately.

## 3. Results

### 3.1 Spatial distribution of traditional villages across Guizhou

#### 3.1.1 Spatial distribution types.

**Fig 2:**
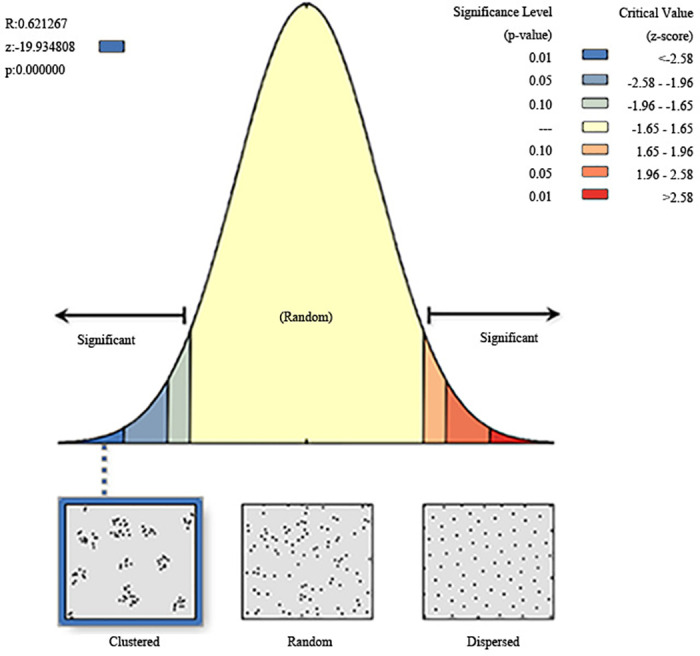
Analysis of the nearest neighbor index for Guizhou’s traditional villages. Note: The base map came from Natural Earth (http://www.naturalearthdata.com/).

For determining the traditional villages’ spatial distribution in Guizhou, we conducted an average nearest neighbor analysis through the use of ArcGIS 10.8. The findings indicate an average inter-village distance of 5311.05 meters, significantly less than the theoretical average distance of 8548.75 meters. The ratio (R) of the actual to theoretical nearest neighbor distance is 0.6212. The test parameters are a z-value of -19.934808 and a p-value below 0.00001. A negative z-value signifies a highly notable aggregation pattern among these villages. Consequently, Guizhou’s traditional villages exhibit a predominantly aggregated spatial distribution (Fig 2).

#### 3.1.2 Spatial distribution equilibrium.

**Fig 3:**
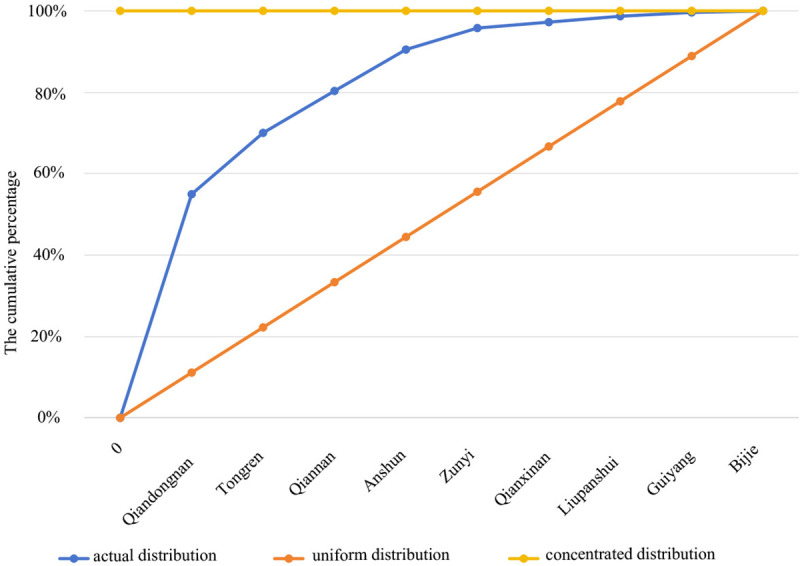
Lorenz curve of spatial distribution.

Both the geographical concentration index and the imbalance index can be utilized to quantify the degree of regional unevenness in the distribution of economy, population, resources, and the like [40]. Regarding the geographical concentration index, Guizhou Province hosts a total of 757 traditional villages distributed across nine cities. The actual geographical concentration index (G) is calculated to be 59.07. Assuming an even distribution, each city would ideally host approximately 84.11 villages, corresponding to a theoretical geographical concentration index ($\begin{array}{c} \bar{G} \end{array}$) of 33.33. $\begin{array}{c} \bar{G} \end{array}$ is conspicuously lower than G. This discrepancy indicates a relative concentration of traditional villages within these cities. The imbalance index (S) is 0.717, suggesting a substantial unevenness in spatial distribution. A Lorenz curve, which was plotted to illustrate this distribution, shows that a significant majority (90.49%) of traditional villages are concentrated in four cities: Qiandongnan Prefecture, Tongren City, Anshun City, and Qiannan Prefecture (Fig 3).

#### 3.1.3 Spatial distribution density.

**Fig 4:**
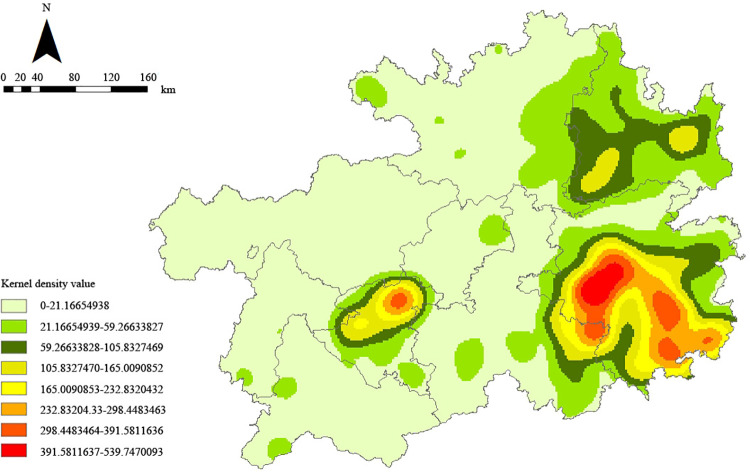
Kernel density map. Note: The base map came from Natural Earth (http://www.naturalearthdata.com/).

Traditional villages’ distribution density in Guizhou has been quantified at 0.43. This metric has also been calculated for traditional villages across the nine cities within the province. Notably, Qiandongnan Prefecture, Tongren City, and Anshun City exhibit distribution densities of 1.37, 0.63, and 0.83, respectively, each surpassing the provincial average. These figures suggest that these three cities constitute the primary agglomeration areas for traditional villages in Guizhou, hosting a significant proportion of these cultural sites. More detailed analysis was conducted on a dataset of 757 traditional villages using the kernel density function within ArcGIS 10.8. This analysis produced the Kernel Density Distribution Characteristic Map of Guizhou’s Traditional Villages (Fig 4), highlighting the “localized high agglomeration and overall low dispersion” spatial distribution pattern, more specifically, the pattern delineates “one highly concentrated area and two secondary density agglomeration areas.” The densest agglomeration, termed the “highly concentrated area,” is located in Qiandongnan, indicating a significant clustering of traditional villages. The “secondary density agglomeration areas” are identified as Anshun City and Tongren City. Furthermore, five prefecture - level cities and autonomous prefectures, such as Zunyi City, Qiannan Buyei and Miao Autonomous Prefecture, and Qianxinan Buyei and Miao Autonomous Prefecture, appear light green on the map, indicating low kernel density values and relatively scattered distribution of traditional villages. The kernel density map visually reflects the unevenly distributed traditional villages in Guizhou Province.

### 3.2. Analysis of determinants of the spatial distribution of traditional villages in Guizhou

#### 3.2.1 Influencing factor selection.

Numerous factors impact traditional villages’ spatial distribution, primarily encompassing elements of the natural environment and socio-cultural dimensions [41], as established by prior research. The natural geographical environment provides the fundamental conditions for the distribution of villages. When selecting locations, traditional villages often favor natural settings that are adjacent to mountains and water bodies. The landscape configuration plays a vital role in aspects such as residents’ production, daily life, and the resistance against natural disasters. Consequently, among the natural - environmental factors, elements like altitude, terrain, climate, and water systems, which are pivotal for village site-selection, spatial layout, and living conditions, have been chosen as independent variables. The social-humanistic environment influences the productive and construction activities carried out by human agglomerations. By enhancing local conditions, it in turn impacts the agglomeration and development of villages. For example, there are relatively more traditional villages in economically underdeveloped regions, while in areas with developed economies and high population densities, their numbers are relatively smaller. Therefore, among the social-environmental factors, indicators closely associated with the inheritance and evolution of traditional-village culture, such as Gross Domestic Product (GDP), per-capita GDP, and the urbanization rate, have been selected as dependent variables. To analyze these influences, this study selects 12 indicators from both natural and socio-cultural categories, detailed in Table 2, to elucidate their effects on traditional villages’ spatial distribution in Guizhou.

**Table 2:** Indicators and calculation formulas for analyzing factors affecting the spatial distribution of traditional villages.

Factor Type	Influencing Factor	Specific Indicator	Calculation Method
Dependent Variable		Traditional Village Concentration (Y)	Traditional Village Average Density in Each Grid
Independent Variable	Natural Environmental Factors	Altitude (X1)	Altitude of Each Grid
		Slope (X2)	Slope of Each Grid
		Aspect (X3)	Aspect of Each Grid
		Temperature (X4)	Annual Average Temperature of Each Grid
		Precipitation (X5)	Annual Average Precipitation of Each Grid
		Hydrological Factors (X6)	Distance from Each Village to the Nearest River
	Socio-cultural Factors	GDP (X7)	Total GDP of Each City
		Per Capita GDP (X8)	Per Capita GDP of Each City
		Urbanization Rate (X9)	Urbanization Rate of Each City
		Population Density (X10)	Population Density of Each City
		Road Network Density (X11)	Road Network Density of Each City
		Ethnic Minority Population Ratio (X12)	Proportion of Ethnic Minority Population in Each City

#### 3.2.2 Natural environmental factors.

##### 1. Elevation

**Fig 5:**
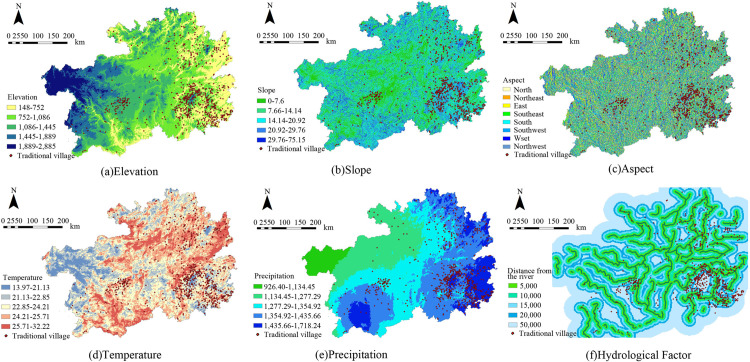
Natural environmental factors and traditional village distribution in Guizhou. Note: The base map came from Natural Earth (http://www.naturalearthdata.com/).

**Fig 6:**
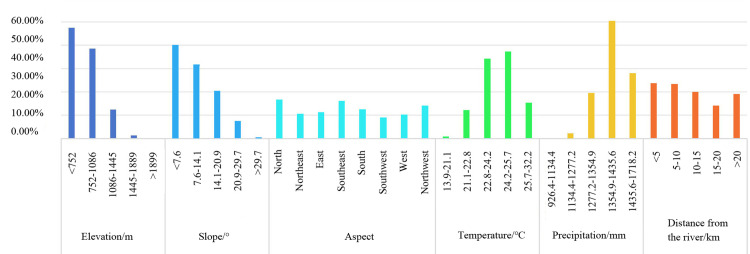
Proportion of traditional villages influenced by natural environmental factors in Guizhou.

Using ArcGIS 10.8, elevation data for each village in Guizhou was extracted. Considering the province’s complex terrain, five elevation categories were established through the natural breaks technique. Analysis shows that the majority of traditional villages, numbering 360 (47.5% of the total), are situated at altitudes below 752 meters. The next largest group, comprising 292 villages (38.5% of the total), is located between 752 and 1086 meters. A sharp decrease in the traditional village count is observed with increasing elevation and decreasing oxygen content, indicating a strong direct link between Guizhou’s traditional village distribution and elevation (Figs 5a and 6).

##### 2. Slope

Slope is a significant topographical factor affecting land use, water resource management, road construction, and agricultural practices. In this study, five slope categories were established through the natural breaks technique. The analysis found 303 villages on slopes less than 7.66°, accounting for 40% of the total. Another 240 villages, representing 31.7% of the total, are located on slopes ranging from 7.66° to 14.14°. Along with the increasing slope, the accessibility of villages diminishes, and the difficulty of housing construction escalates, and the count of traditional villages declines sharply, demonstrating a strong positive correlation between the slope and the distribution of Guizhou’s traditional villages (Figs 5b and 6).

##### 3. Aspect

The aspect of a village affects its exposure to sunlight, solar radiation, precipitation, and temperature, which in turn can influence the village’s spatial layout. However, due to the overall scarcity of sunshine across Guizhou Province, the orientation of houses does not adhere strictly to the south - facing requirement [42]. Guizhou’s traditional village distribution changes notably in various aspects, with a predominance on shaded slopes (north, northeast, east, and southeast), which house 412 villages or 54.42% of the total. Specifically, the north and southeast-facing slopes are the most populated, containing 32.62% of the province’s traditional villages. Conversely, sunny slopes (south, southwest, west, and northwest) host 345 villages, which constitute 45.58% of the total (Figs 5c and 6).

##### 4. Temperature

Temperature is a critical factor in agricultural productivity, directly affecting crop growth, development, and yield. The prevailing long-term temperature conditions largely dictate the spatial distribution of traditional villages. Thus, we employed “annual mean temperature” as an indicator. By overlaying the annual mean temperatures of Guizhou for 2023 with the locations of traditional villages using ArcGIS, we observed that 542 villages have annual mean temperatures ranging from 22.8°C to 25.7°C, representing 71.59% of the total. Higher mean temperatures occur in the southeast, southwest, and north of Guizhou, yet these conditions do not appear to hinder traditional village distribution. Conversely, when the mean temperature over the year falls below 21.1°C, there are much fewer traditional villages (Figs 5d and 6).

##### 5. Precipitation

Precipitation, alongside temperature, serves as a vital indicator of local climatic conditions. It affects climate humidity, soil moisture levels, and the development of river networks. In this study, 593 traditional villages are primarily located in areas receiving annual rainfall between 1354.9 and 1718.2 millimeters, accounting for 78.3% of the total. This range of precipitation supports optimal agricultural conditions and somewhat mitigates the risk of flooding. Conversely, traditional villages do not exist in regions where the yearly rainfall falls below 926.4 millimeters because conditions are unfavorable for agricultural production and residents’ livelihoods.(Figs 5e and 6).

##### 6. Hydrological Factors

Water, essential for life, has a substantial effect on traditional village distribution, often determined by the availability and distribution of water resources. In Guizhou, which is intersected by numerous rivers including those within the Yangtze River Basin and the Pearl River Basin, our study utilized ArcGIS 10.8’s “buffer zone analysis” to categorize proximity to major rivers into 5 zones: 0–5, 5–10, 10–15, 15–20 km, and greater than 20 km. The analysis indicates a relatively even distribution of villages across these zones, with counts of 179, 176, 151, 106, and 145 respectively, suggesting a low dependency on proximal river water sources. Notably, some villages in southeastern Guizhou, near Leigong Mountain and more than 20 km from major rivers, have clustered together, relying on wells and streams for their water supply (Figs 5f and 6).

#### 3.2.3 Guizhou traditional village distribution influenced by socio-cultural factors.

##### 1. GDP

**Fig 7:**
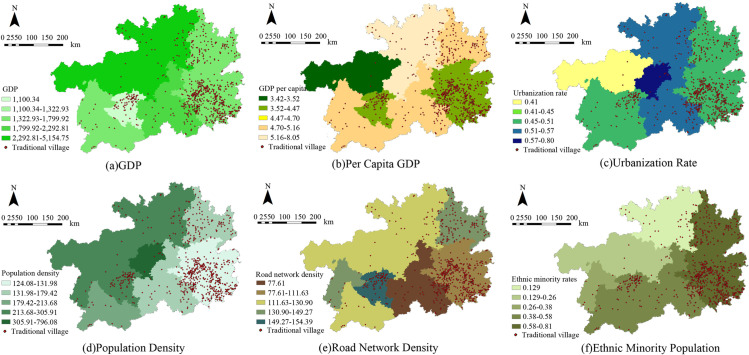
Distribution of sociocultural elements and traditional villages in Guizhou. Note: The base map came from Natural Earth (http://www.naturalearthdata.com/).

**Fig 8:**
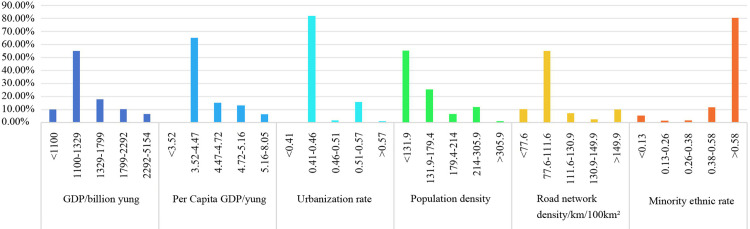
Proportion of Traditional Villages Influenced by Sociocultural Factors in Guizhou.

Gross Domestic Product (GDP) has a dual impact on traditional village distribution. On the one hand, regional economic development provides more opportunities, resources, funds and policy support for village development. On the other hand, the long - standing drawbacks in China’s system of evaluating official performance based on GDP have led to a weak awareness of traditional village protection in many regions, thereby accelerating the disappearance of traditional villages. In 2023, there was a large GDP gap among various cities in Guizhou Province. The GDP of the provincial capital, Guiyang, was 515.475 billion yuan, while Anshun ranked the lowest, with only 110.034 billion yuan [43]. After analyzing the connection of traditional villages and various indicators across nine cities in Guizhou, we confirm a clear correlation between village distribution and these cities’ economic development levels. The study categorized GDP into five groups using the natural breaks method. Traditional villages are predominantly present in areas with lower GDP, with the greatest concentration (414 villages, accounting for 54.6%) situated in regions where GDP ranges from 110 billion to 132.9 billion yuan. This indicates that regions with underdeveloped economies are more conducive to the preservation of traditional villages. The count of traditional villages significantly diminishes outside this range (Figs 7a and 8).

##### 2. Per Capita GDP

The influence of per capita GDP on the distribution of traditional villages is akin to that of overall GDP. However, per capita GDP incorporates the population quantity indicator. Consequently, cities with a lower per capita GDP are more backward, exerting a more pivotal role in the spatial distribution, quantity, and conservation of traditional villages. In 2023, among the various cities in Guizhou Province, the provincial capital, Guiyang, boasted the highest per capita GDP, reaching 81,670 yuan, while Bijie had the lowest, merely 34,200 yuan [43]. In contrast to overall GDP, per capita GDP is more influential in shaping the spatial distribution and determining traditional village count. For this analysis, five per capita GDP groups were established through the natural breaks approach. The category ranging from 35,200–44,700 Chinese yuan includes the largest village count. Notably, 492 villages take up 64.9%. The prevalence of traditional villages decreases sharply outside this range, mirroring the general patterns observed in the GDP analysis (Figs 7b and 8).

##### 3. Urbanization rate

The urbanization rate serves as an indicator of how urbanization levels influence traditional village distribution. In 2023, among all cities in Guizhou Province, the provincial capital, Guiyang, exhibited the highest urbanization rate, reaching 0.80, while Bijie had the lowest, with a mere 0.42 [44]. Most of Guizhou’s traditional villages, totaling 618, exist in cities where the rates of urbanization lie between 0.41 and 0.46, comprising 81.6% of all villages. It is noteworthy that the connection of the urbanization rate and the prevalence of traditional villages is not strictly inverse, as evidenced by the presence of 118 villages (20.5% of the total) in areas with urbanization rates ranging from 0.51 to 0.57. This suggests that the urbanization rate is one of the factors influencing the distribution of traditional villages, yet it is not the sole determinant (Figs 7c and 8).

##### 4. Population density

Population density, a key demographic factor, significantly impacts the way traditional villages are developed and transformed. In 2023, among various cities and prefectures in Guizhou Province, Guiyang, the provincial capital, recorded the highest population density, reaching 797 people per square kilometer, while Qiandongnan Prefecture had the lowest, with only 124 people per square kilometer [45]. In regions with the population density < 131.9 people per square kilometer, the count of traditional villages stands at 414, representing 54.6% of the total. Conversely, areas with a population density between 131.9 and 179.4 people per square kilometer contain 194 villages, representing 25.6% of the total. This distribution suggests a clear connection of population density with the presence of traditional villages, with fewer villages in more densely populated areas and more in less populated ones (Figs 7d and 8).

##### 5. Road network density

Transportation accessibility, measured by road network density, crucially determines traditional village spatial distribution and preservation. Utilizing ArcGIS 10.8, road densities were calculated for each city or prefecture. The majority of traditional villages in Guizhou are present in areas with moderate to low transportation infrastructure. Specifically, the highest concentration of villages (416, accounting for 54.9%) is found in areas with road network densities ranging from 77.6 to 111.6 kilometers per square kilometer. This underdevelopment in transportation infrastructure contributes significantly to the preservation of traditional villages (Figs 7e and 8).

##### 6. Ethnic minority population ratio

Guizhou Province is home to a significant concentration of ethnic minorities making up 37.9% of the total population [46]. Notably, the Miao, Buyi, Dong, and Shui ethnic groups have the largest populations nationally. The traditional village spatial distribution correlates strongly with the presence of ethnic minority populations in the study area. A total of 608 traditional villages, representing 80.3% of all villages, have an ethnic minority population ratio exceeding 0.58. Collectively, areas with higher ratios of ethnic minorities usually have more traditional villages (Figs 7f and 8).

### 3.3 Detection of factors influencing the distribution of traditional villages

#### 3.3.1 Single-factor detection.

In this research, twelve sets of data were identified as influential factors for traditional villages, classified as follows: altitude (X1), slope (X2), aspect (X3), temperature (X4), precipitation (X5), distance from rivers (X6), GDP (X7), per capita GDP (X8), urbanization rate (X9), population density (X10), road network density (X11), and ethnic minority population ratio (X12). Initially, ArcGIS was utilized to transform the point features of traditional villages into kernel density data (Y), which was treated as the dependent variable and quantified numerically. The twelve influencing factors, defined as independent variables, were initially numerical and were categorized through classification methods into six groups by virtue of the natural breaks method. The fishnet tool was employed to overlay and extract data points onto a grid for subsequent analysis. The influence of each factor on traditional village distribution was then quantitatively assessed using Geodetector tool in EXCEL. For the single factor X influencing the spatial differentiation of traditional villages at the city-level in Guizhou Province, the closer the q-value is to 1, the more significant the explanatory power. With the exception of temperature (X4), whose p-value is greater than 0.05 and thus lacks representativeness, the factors were ranked in terms of their influence from highest to lowest as follows: road network density, GDP, ethnic minority population ratio, per capita GDP, urbanization rate, population density, precipitation, altitude, slope, distance from rivers, temperature, and aspect (Table 3). The analysis confirms that socio-cultural factors more dramatically impact traditional villages’ distribution versus natural environmental factors.

**Table 3:** Results of factor detection for the distribution of traditional villages in Guizhou.

	X1	X2	X3	X4	X5	X6	X7	X8	X9	X10	X11	X12
q statistic	0.072	0.016	0.002	0.005	0.165	0.012	0.361	0.258	0.182	0.170	0.372	0.264
p value	0.000	0.003	0.031	0.373	0.000	0.008	0.000	0.000	0.000	0.000	0.000	0.000

Traditional villages’ spatial variability is shaped by a complex interplay between natural geographical conditions and socio-cultural factors. The natural geographical environment provides the foundational framework for village distribution, while socio-cultural elements influence the overall layout and lead to local variations in spatial distribution [47]. This observation contrasts with prior studies on traditional villages in southwestern China, predominantly highlighting the significant influence of natural geographical factors [48]. However, research focused on Guizhou Province indicates that socio-cultural factors have a more dominant influence relative to natural geographical conditions, suggesting that the relatively low levels of economic development in these villages heighten their sensitivity to socio-cultural changes. Economic shifts driven by urbanization may influence surrounding rural areas, but in economically advanced cities, rural populations are often drawn to urban centers, resulting in rural-to-urban migration, which can lead to village depopulation and even disappearance. Among the twelve influencing factors examined, six key socio-cultural elements rank in the top positions, underscoring the significant impact of urbanization on traditional villages. Conversely, natural environmental factors exhibit relatively less explanatory power in this study, with factors including precipitation, altitude, slope, distance from rivers, temperature, and aspect ranking lowest. Initially, natural factors like terrain and proximity to water systems were crucial in early village settlements, which were predominantly agricultural and often situated on flat terrain near rivers with favorable temperatures. However, in recent decades, with ongoing economic growth, natural environmental factors have exerted a diminished influence on traditional village preservation and enhancement, and their sensitivity to these factors has weakened over time.

#### 3.3.2 Detection of interaction factors.

Multiple factors collectively shape the spatial distribution of traditional villages, affecting their layout and location. To further explore the extent of interaction among these factors, we adopted the Geodetector tool for evaluating the impact of pairwise interactions on spatial distribution. The findings suggest that two interacting factors exert a more obvious influence versus individual factors alone, with the q-value of each factor increasing under interaction. The nature of these interactions is predominantly characterized by nonlinear enhancement. Specifically, 31 dual-factor interactions exhibit enhancement, and 41 show nonlinear enhancement, both surpassing the impact of single factors. The factors exhibiting the greatest pairwise interactive influence include altitude (X1), GDP (X7), per capita GDP (X8), urbanization rate (X9), population density (X10), road network density (X11), and ethnic minority population ratio (X12) (Table 4).

**Table 4:** Results of interaction detection for the distribution of traditional villages in Guizhou. (The bold font indicates dual-factor enhancement of interaction, while the normal font represents nonlinear enhancement.).

	X1	X2	X3	X4	X5	X6	X7	X8	X9	X10	X11	X12
X1	0.0721											
X2	0.0990	0.0159										
X3	0.0813	0.0440	0.0023									
X4	0.1514	0.0662	0.0317	0.0048								
X5	0.2552	0.2136	0.1905	0.2105	0.1653							
X6	0.1118	0.0634	0.0292	0.0591	0.1953	0.0122						
X7	**0.4265**	0.4017	0.3832	0.4003	**0.3941**	0.4393	**0.3614**					
X8	0.3643	0.3060	0.2765	0.2904	**0.4149**	0.3131	**0.3855**	**0.2579**				
X9	**0.2164**	0.1995	0.1972	0.2133	**0.2292**	0.2020	**0.3664**	**0.3857**	0.1818			
X10	**0.2286**	0.2080	0.1811	0.2030	**0.3312**	0.2236	**0.3857**	**0.3857**	**0.3857**	**0.1701**		
X11	**0.4355**	0.4123	0.3935	0.4138	**0.4060**	0.4485	**0.3733**	**0.3857**	**0.3857**	**0.3857**	**0.3720**	
X12	**0.3024**	0.2959	0.2809	0.3048	**0.3007**	0.3368	**0.3857**	**0.3857**	**0.2644**	**0.3856**	**0.3857**	**0.2637**

## 4. Discussion

### 4.1 The formation process of the spatial distribution pattern of traditional villages

Historically, the factors affecting the traditional village spatial distribution have evolved significantly. Before the Yuan Dynasty, regions like Guizhou were expansive and sparsely populated, characterized by a rudimentary mode of rural production with agriculture as the predominant industry [49]. During this period, factors including proximity to rivers, levels of precipitation, and temperature played critical roles in the early enhancement of traditional villages. The complex terrain and notable elevation differences within the region prompted farmers, motivated by the need for arable land, to settle in areas of higher altitude and marked topographic relief, thus maximizing the available flat land. Clearly, natural environmental factors were pivotal for traditional villages in terms of the initial establishment and spatial organization.

With the onset of the Yuan Dynasty, ethnic culture primarily impacted the spatial distribution and evolving patterns with regard to traditional villages across various regions. Population growth in Guizhou was largely driven by the migration of ethnic minorities. These groups, seeking to preserve their cultural identity and for defensive reasons, often settled in steep mountainous areas, as evidenced by the high concentration of Miao villages in Leishan. Additionally, ethnic distinctions sometimes fostered separation; for example, the migration of Han people during the Ming Dynasty led ethnic minorities to maintain distance, resulting in a pattern where ethnic minority villages were situated far from “tunbao” (fortified villages) [50].

Following the Qing Dynasty, rapid socio-economic advancements significantly increased population mobility between urban and rural areas, remarkably weakening the vitality of traditional villages. In Guizhou, where traditional villages generally lagged economically, the advance of industrial development transformed production modes and gradually diminished the living spaces of traditional culture and villages. As a result, traditional villages were more commonly located in impoverished areas distant from urban centers [51]. However, economic development also had beneficial effects. For instance, the government implemented various policies aimed at protecting and developing traditional villages, utilizing economic, cultural, and ecological resources to foster the growth of traditional cultural industries and tourism, thus enhancing the development of these villages. Moreover, the availability of transportation infrastructure could more greatly facilitate agricultural trade and rural tourism, effectively stimulating local development. Consequently, villages situated near roads often displayed more prosperous scenes.

In conclusion, the natural environment initially established the foundational distribution framework pertaining to traditional villages in the region. Ethnic and cultural factors later emerged as dominant forces, amplifying the disparities in long-term distribution patterns. Throughout the urbanization process, socio-cultural factors have ascended as the primary drivers, playing a decisive role in the sustainable enhancement of traditional villages in the region. The complex interplay and influence of these various factors have profoundly shaped traditional village distribution pattern in the region.

### 4.2 Strategies for the protection and enhancement of traditional villages

Upon examining traditional villages in Guizhou in terms of the distribution characteristics and underlying factors, it becomes clear that strategies for their preservation and enhancement must judiciously balance development with conservation. Initially, strategies should incorporate considerations of natural environmental and ethnic and cultural factors to preserve authentic traditional villages. Subsequently, economic aspects, particularly those related to agriculture and tourism, need to be addressed to sustain their development momentum. Drawing from the discussion above, four principal areas of focus are proposed for traditional village protection and enhancement in Guizhou:

#### 1. Establishing regional traditional village identities by integrating local ethnic characteristics

Guizhou has the highest number of ethnic groups in China [46]. Ethnic minority population proportion significantly influences traditional village distribution (q = 0.264). Traditional villages in Guizhou are notably distinguished by their rich ethnic resources, setting them apart on a national level. The nearest neighbor index (R) for these villages is 0.6212, hinting a high concentration in ethnic minority areas. To protect these villages, it is imperative to prioritize the preservation of diverse ethnic cultures, including traditional customs, artistic expressions, and intangible cultural heritage, such as heritage-focused tourism, educational programs in culture, and events dedicated to heritage. At the municipal level, establishing traditional village conservation areas can foster local traditional village growth and boost their attractiveness. In creating traditional village identities, local cultural and architectural characteristics must be considered to generate an image that resonates with local features, thereby boosting their appeal and influence.

#### 2. Improving village infrastructure to enhance accessibility

Guizhou’s landscape is predominantly plateau and mountainous, with mountainous regions constituting 84% of the province and plateau-mountainous areas comprising 94% [52]. Terrain critically influences the traditional village distribution in Guizhou, with the slope being a primary natural environmental determinant (q = 0.016). The mountainous terrain poses significant transportation challenges, complicating the construction of roads, bridges, and other infrastructure. Road network density also crucially affects the traditional village distribution (q = 0.372). Establishing a comprehensive transportation network and infrastructure is essential for traditional village protection and enhancement. Enhancing transportation improves traditional villages’ connections and reachability, attracting more tourists and investments, and fostering their development which supports the preservation and transmission of traditional cultures and historical heritage. Therefore, to achieve the sustainable and stable development of traditional villages in Guizhou, a comprehensive consideration of terrain and road - network density factors is essential. In the policies designed to support these traditional villages, intensified investment in infrastructure construction and the enhancement of transportation networks should be prioritized. This approach would not only address the geographical and connectivity challenges but also lay a solid foundation for the long - term viability and prosperity of these culturally and historically significant settlements.

#### 3. Implementing targeted, concentrated protection and utilization based on local conditions

According to Kernel density analysis, traditional villages in Guizhou are characterized by a distribution pattern featuring “one highly concentrated area and two secondary density clusters.” Accordingly, protection and utilization efforts should focus on counties, cities, districts, and areas with a high concentration of traditional villages, particularly those that have demonstrated significant achievements in their preservation and enhancement. This approach advocates expanding protection efforts from individual points to broader areas. For traditional villages that possess unique characteristics and advantages, development plans such as “one village, one product” can be formulated. These plans should be tailored based on the specific conditions and resource advantages of each village, thereby determining the direction of specialized industries for each. This strategy enables the implementation of differentiated development and the formulation of “one village, one industry” strategies. By treating traditional villages as developmental nodes and establishing developmental orientations that reflect actual conditions, resource advantages can be fully utilized to promote the layout of specialized industries. This approach will facilitate the dedicated safeguarding and application of Guizhou’s traditional villages, emphasizing their holistic value and coordinating various resources to form a unified effort towards rural revitalization and the preservation and development of exceptional traditional cultures.

#### 4. Industrial linkage, combining protection and development.

The dual principles of developing while protecting and protecting while developing should be consistently applied to traditional village projects. As previously discussed, among determinants of traditional village spatial distribution, social and human factors including GDP, per capita GDP, population density, urbanization rate, ethnic minority proportion, and road network density exhibit relatively high explanatory power. Their connection with the count of traditional villages is neither linearly increasing nor decreasing but exhibits the most significant impact within a specific range. Preserving and protecting traditional villages requires identifying an appropriate balance within the socio-economic development spectrum. The development of the economy is a double-edged sword for the protection of traditional villages. On one hand, it can boost the regional economy and the tourism industry, providing traditional villages with more opportunities, resources, funds, and policy support for their development. However, if there is a one-sided pursuit of economic value, it will severely undermine the authentic cultural characteristics and the original ecological natural environment of traditional villages, accelerating the disappearance of these traditional settlements. For instance, promoting sustainable and eco-friendly farming methods that are tailored to the distinct ecological features of the area, including the encouragement of organic farming, traditional crop growing, and agritourism, can enhance local agriculturists’ financial conditions while protecting the environment. This, in turn, stimulates government financial and policy support, thereby partially promoting the protection of traditional villages. Ultimately, this strategy assists in organically integrating traditional village protection with tourism development.

## 5. Conclusions

1. The present study examines 757 traditional villages’ spatial distribution and influencing factors across Guizhou, China, utilizing spatial autocorrelation, geodetector approaches, kernel density estimation, and nearest neighbor analysis. This investigation has led to the formulation of conservation strategies based on the derived insights. The key findings are summarized below:
2. Traditional villages in Guizhou present a clustered spatial distribution, characterized by “one highly concentrated area” in southeast Guizhou and “two secondary density clusters” in Anshun and Tongren.
3. Natural and socio-cultural factors can both affect traditional villages’ spatial distribution. Natural factors such as elevation, slope, proximity to rivers, temperature, and precipitation set the foundational framework for village distribution, while socio-cultural factors, including economic indicators (GDP, urbanization rate, and road network density, etc.) and ethnic minority populations, play a critical role in shaping spatial patterns. Compared with natural factors, socio - cultural factors more remarkably affect the village distribution. The interplay of these factors shows nonlinear enhancement, suggesting a complex and synergistic influence on spatial patterns.
4. The formation process of the above distribution patterns is mainly attributed to population migration, the requirements of wars, and economic development. The immigration of ethnic minorities directly contributed to the formation of massive traditional ethnic minority villages in Guizhou. The demands of wars led to the emergence of some military - defensive villages. Meanwhile, economic development accelerated the urban-rural area population mobility, making villages extensively replicated and differentiated.
5. Strategies for preserving and enhancing traditional villages should focus on: (1) developing regional identities that integrate local ethnic characteristics, (2) improving village infrastructure to enhance accessibility, (3) implementing targeted protection and development plans considering local conditions, and (4) industrial linkage, combining protection and development.

This paper highlights traditional villages’ spatial distribution characteristics in Guizhou Province from a macroscopic perspective. By employing digital means, it dissects the influencing factors underlying the formation of traditional villages. The findings presented herein hold significant reference value for the conservation and development of traditional villages. Despite providing helpful insights into traditional villages’ spatial distribution in Guizhou and proposing conservation strategies, several limitations in the study require further investigation. The spatial distribution characteristics of traditional villages in Guizhou Province result from the combined effects of natural, social, and humanistic factors, and the interactions among these factors are highly intricate. The present study cannot comprehensively encompass all influencing factors. Future research should aim to enhance the comprehensiveness of influencing factors, particularly those related to socio-economic and cultural dimensions. Additionally, tailored micro-level analyses are needed to comprehensively acquire data on historical evolution, living habits, architectural attributes, industrial development, artistic value, and the physical environment of villages. This approach aims to better unveil the formation mechanisms and driving forces of traditional villages, thereby highlighting the individual disparities among them, supporting more nuanced policy-making that effectively balances cultural preservation with rural revitalization and development.

## Supporting information

S1 Dataset Dataset of 757 traditional villages in Guizhou Province, including village name, location, topographic, climatic, hydrological, and socio-economic attributes. (RAR)

S2 Dataset Regional climatic and economic indicators at the county level used for contextual analysis. (RAR)

S3 File Descriptions of all data fields and their respective sources, including URLs and reference documents. (RAR)
